# Laparoscopic Versus Open Appendectomy in Complicated and Uncomplicated Appendicitis in Adults: A Two-Year Single-Center Retrospective Cohort Study

**DOI:** 10.7759/cureus.92258

**Published:** 2025-09-14

**Authors:** Parin Y Patel, Ronak Rathod, Milind K Akhani

**Affiliations:** 1 General Surgery, Health1 Super Speciality Hospital, Ahmedabad, IND; 2 Surgical Gastroenterology, HPB Surgery and Liver Transplantation, Health1 Super Speciality Hospital, Ahmedabad, IND

**Keywords:** acute appendicitis, complicated appendicitis, laparoscopic appendectomy, length of stay, open appendectomy, postoperative complications

## Abstract

Introduction: Laparoscopic appendectomy is now widely regarded as the preferred approach for acute appendicitis. However, its role in complicated appendicitis remains debated, particularly in settings where open surgery is still frequently performed. This study aimed to compare outcomes of laparoscopic and open appendectomy in both complicated and uncomplicated appendicitis.

Methods: We conducted a retrospective, single-center cohort study of adult patients (≥18 years) who underwent appendectomy between January 1, 2023, and December 31, 2024, at the Department of General Surgery, Health One Super Speciality Hospital, Ahmedabad, India. Patients undergoing concomitant major abdominal procedures, interval appendectomy, or with incomplete records were excluded. Outcomes assessed included operative time, postoperative complications, reoperation, and length of hospital stay.

Results: Of 337 patients, 282 were included in the final analysis (248 laparoscopic, 34 open). Median operative time was longer in laparoscopic surgery (60 vs 50 minutes; p=0.020). Drains were placed only in two complicated open cases. Overall, postoperative ileus was more frequent in the open group (14.7% vs 4.0%; p=0.020). Surgical site infections (SSIs) were more common after open appendectomy, though differences were not statistically significant. Median hospital stay was significantly shorter for laparoscopic cases (four vs six days; p<0.001). In complicated appendicitis, laparoscopic appendectomy was associated with a lower ileus rate (7.1% vs 26.3%; p=0.025) and shorter stay (five vs seven days; p<0.001). In uncomplicated appendicitis, complication rates were low in both groups, but laparoscopic surgery reduced hospital stay (three vs five days; p<0.001).

Conclusion: Laparoscopic appendectomy demonstrated favorable outcomes compared with open surgery, particularly in complicated appendicitis, with reduced ileus and shorter hospitalization. These findings support laparoscopic surgery as the preferred approach for both complicated and uncomplicated appendicitis in adult patients.

## Introduction

Acute appendicitis is one of the most frequent surgical emergencies worldwide, with a lifetime risk of approximately 7-8% [1]. Despite being a common condition, the optimal surgical management of appendicitis continues to evolve. For decades, open appendectomy was regarded as the standard procedure, and it remains widely performed in many parts of the world [2]. The advent of laparoscopic appendectomy in the 1980s revolutionized the management of appendicitis, offering patients a minimally invasive alternative [3].

Several studies have reported the advantages of the laparoscopic approach, including reduced postoperative pain, lower wound infection rates, shorter hospital stays, and faster return to daily activities [4]. In addition, laparoscopy allows for better visualization of the peritoneal cavity, making it possible to identify unexpected pathology and manage concomitant intra-abdominal conditions. However, concerns persist regarding increased operative times, higher costs, the need for specialized equipment, and the potential risk of intra-abdominal abscess formation, especially in cases of perforated or gangrenous appendicitis [5].

Complicated appendicitis, characterized by perforation, gangrene, abscess, or phlegmon, presents additional management challenges. Such cases are associated with higher morbidity, longer hospitalization, and greater resource utilization compared to uncomplicated appendicitis [6]. The debate over whether laparoscopic appendectomy offers the same benefits in complicated appendicitis as it does in uncomplicated cases remains unresolved. Some evidence suggests laparoscopy may reduce wound complications even in complicated cases, while other studies caution about higher intra-abdominal infection rates [5,6].

The choice between laparoscopic and open appendectomy also varies widely across regions, influenced by surgeon preference, patient presentation, and institutional resources. In high-income countries, laparoscopy is increasingly the default approach, whereas in resource-limited settings, open appendectomy is still commonly performed due to equipment availability and cost considerations [2]. This variation underscores the importance of context-specific data to guide surgical decision-making.

While numerous studies from Western populations have addressed this question, comparative data from the Indian subcontinent remain relatively sparse. Given differences in patient demographics, disease presentation, and healthcare infrastructure, findings from other regions may not be fully generalizable. Local evidence is therefore essential to determine whether the advantages of laparoscopic appendectomy translate into better outcomes in both complicated and uncomplicated cases within this setting.

This study aimed to compare short-term outcomes of laparoscopic and open appendectomy in adults with acute appendicitis. The primary outcome was postoperative complications, including surgical site infection (SSI), postoperative ileus, postoperative bleeding, and reoperation within 30 days. Secondary outcomes included operative time, drain use, and length of hospital stay. Because outcomes may differ substantially between complicated and uncomplicated appendicitis, analyses were performed both overall and stratified by disease severity.

## Materials and methods

Study design and setting

This was a retrospective, single-center cohort study conducted at Health One Super Speciality Hospital, Ahmedabad, Gujarat, India, a tertiary care referral center. The study included patients who underwent appendectomy between 1 January 2023 and 31 December 2024. Ethical approval was obtained from the Institutional Ethics Committee, and the requirement for informed consent was waived as only anonymized medical records were reviewed.

Study population

All adult patients aged 18 years and above who underwent appendectomy for acute appendicitis during the study period were included. Both laparoscopic and open appendectomy cases were analyzed. Patients who underwent concomitant major abdominal procedures, those who had appendectomy for non-acute indications such as interval surgery, and those with incomplete medical records were excluded.

Definitions

Laparoscopic appendectomy was generally performed using a standard three-port technique with endoloop ligation of the appendiceal stump. Open appendectomy was performed via a standard right lower quadrant incision (McBurney or Lanz) with simple ligation of the stump.

Complicated appendicitis was defined as intraoperative or histopathological evidence of perforation, gangrene, abscess, or phlegmon. Uncomplicated appendicitis was defined as an inflamed or suppurative appendix without these features.

SSI was classified as superficial incisional (skin and subcutaneous tissue), deep incisional (fascial and muscle layers), and organ/space infection (e.g., intra-abdominal abscess), assessed up to 30 days after surgery.

Postoperative ileus was defined as intolerance to oral intake or failure to pass flatus/stool beyond postoperative day three, with or without abdominal distension, requiring nasogastric decompression.

Data collection

Data were extracted retrospectively from medical records, operative notes, and discharge summaries using a structured proforma. Information collected included demographic details (age and sex), clinical presentation (duration of symptoms and preoperative laboratory findings where available), operative details (surgical approach, operative time, drain placement, intraoperative findings), and postoperative outcomes.

Outcome measures

The primary outcome was postoperative complications, defined as SSI (superficial, deep, or organ-space), postoperative ileus, postoperative bleeding, and reoperation within 30 days. Secondary outcomes included operative time, drain use and duration, and hospital stay. Outcomes were analyzed both in the overall cohort and separately for patients with complicated and uncomplicated appendicitis.

Statistical analysis

Continuous variables, such as operative time and length of hospital stay, were expressed as median with interquartile range (IQR) and compared between groups using the Mann-Whitney U test. Categorical variables, including postoperative complications, were presented as frequencies and percentages. Comparisons between the laparoscopic and open appendectomy groups were performed usingFisher’s exact test. All tests were two-tailed, and a p-value of <0.05 was considered statistically significant. Statistical analyses were carried out using Statistical Product and Service Solutions (SPSS, version 25; IBM SPSS Statistics for Windows, Armonk, NY).

## Results

**Figure 1:**
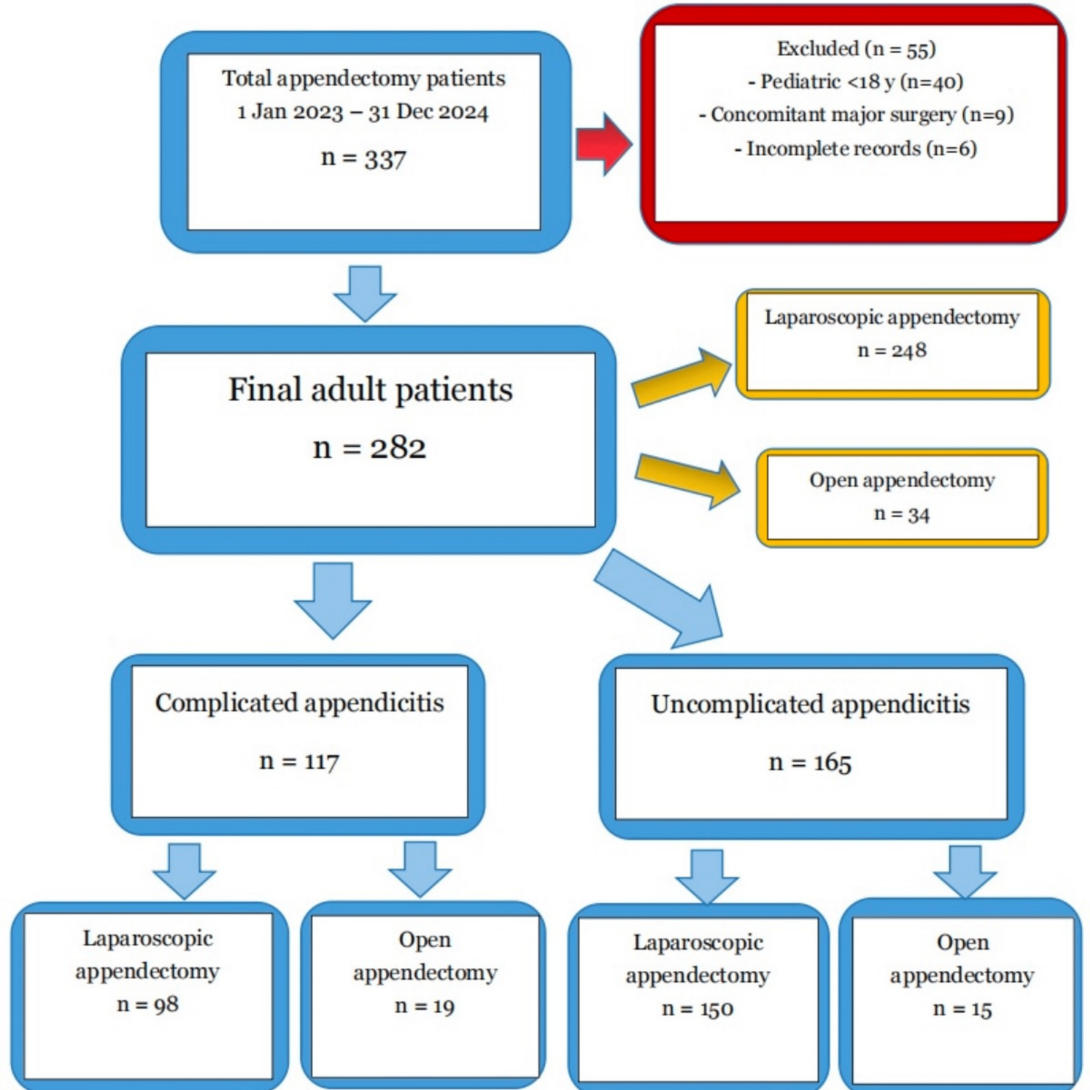
Study flow diagram showing patient selection, exclusions, and final distribution by surgical approach and appendicitis type.

A total of 337 patients underwent appendectomy between 1 January 2023 and 31 December 2024. Of these, 55 were excluded (40 patients aged <18 years, nine with concomitant major abdominal procedures, and six with incomplete records), leaving 282 adults in the final analysis. Among them, 248 (87.9%) underwent laparoscopic appendectomy, and 34 (12.1%) underwent open appendectomy (Figure 1).

Baseline characteristics are shown in Table 1. The median age was 34 years in the laparoscopic group and 36 years in the open group (p=0.427). Sex distribution was similar (male: 58.9% vs 61.8%; p=0.747). A higher proportion of complicated appendicitis was observed in the open group compared with the laparoscopic group (55.9% vs 39.5%), though this difference was not statistically significant (p=0.094).

**Table 1:** Baseline characteristics of adults undergoing laparoscopic and open appendectomy. N = total number of patients in each group; n = number of patients with the specified characteristic; IQR = interquartile range

Characteristic	Laparoscopic (N=248)	Open (N=34)	p-value
Age, years, median (IQR)	34 (26–44)	36 (27–48)	0.427
Male, n (%)	146 (58.9)	21 (61.8)	0.747
Female, n (%)	102 (41.1)	13 (38.2)	0.747
Complicated appendicitis, n (%)	98 (39.5)	19 (55.9)	0.094
Uncomplicated appendicitis, n (%)	150 (60.5)	15 (44.1)	0.094

Operative details are summarized in Table 2. Median operative time was longer in laparoscopic appendectomy (60 minutes) compared with open appendectomy (50 minutes; p=0.020). Drains were not used in laparoscopic cases but were placed in two open procedures (5.9%), both involving complicated appendicitis (p=0.014). The median duration of drainage was three days.

**Table 2:** Operative details of laparoscopic and open appendectomy. N = total number of patients in each group; n = number of patients with the specified characteristic; IQR = interquartile range *Significant

Variable	Laparoscopic (N=248)	Open (N=34)	p-value
Operative time, min, median (IQR)	60 (50–75)	50 (40–65)	0.020*
Drain placed, n (%)	0 (0.0)	2 (5.9)	0.014*
Drain duration, days, median (IQR)	–	3 (2–3)	–

Overall postoperative outcomes are presented in Table 3. Superficial, deep, and organ-space SSIs occurred more often after open surgery, although differences were not significant. Ileus was more frequent in open cases (14.7% vs 4.0%; p=0.020). Postoperative bleeding was uncommon (2.9% vs 1.6%; p=0.447). Median length of stay was six days for open and four days for laparoscopic cases (p<0.001). Reoperation within 30 days occurred in one open (2.9%) and five laparoscopic patients (2.0%; p=1.000).

**Table 3:** Overall postoperative outcomes following laparoscopic and open appendectomy. N = total number of patients in each group; n = number of patients with the specified characteristic; SSI = surgical site infection; IAA = intra-abdominal abscess; IQR = interquartile range *Significant

Outcome	Laparoscopic (N=248)	Open (N=34)	p-value
Superficial SSI, n (%)	11 (4.4)	4 (11.8)	0.092
Deep SSI, n (%)	6 (2.4)	2 (5.9)	0.249
Organ-space SSI / IAA, n (%)	9 (3.6)	3 (8.8)	0.165
Postoperative ileus, n (%)	10 (4.0)	5 (14.7)	0.020*
Postoperative bleeding, n (%)	4 (1.6)	1 (2.9)	0.447
Length of stay, days, median (IQR)	4 (3–5)	6 (5–7)	<0.001*
Reoperation ≤30 days, n (%)	5 (2.0)	1 (2.9)	1.000

Outcomes in complicated appendicitis are shown in Table 4. SSI occurred in 15.8% of open and 8.2% of laparoscopic cases (p=0.360). Ileus was significantly more frequent in open cases (26.3% vs 7.1%; p=0.025). Median hospital stay was longer after open surgery (seven vs five days; p<0.001). Reoperation rates were low and comparable (5.3% vs 3.1%; p=0.620).

**Table 4:** Postoperative outcomes in patients with complicated appendicitis by surgical approach. N = total number of patients in each group; n = number of patients with the specified characteristic; SSI = surgical site infection; IAA = intra-abdominal abscess; IQR = interquartile range *Significant

Outcome	Laparoscopic (N=98)	Open (N=19)	p-value
Superficial SSI, n (%)	8 (8.2)	3 (15.8)	0.360
Deep SSI, n (%)	4 (4.1)	2 (10.5)	0.280
Organ-space SSI / IAA, n (%)	7 (7.1)	3 (15.8)	0.210
Postoperative ileus, n (%)	7 (7.1)	5 (26.3)	0.025*
Postoperative bleeding, n (%)	2 (2.0)	1 (5.3)	0.430
Length of stay, days, median (IQR)	5 (4–6)	7 (6–8)	<0.001*
Reoperation ≤30 days, n (%)	3 (3.1)	1 (5.3)	0.620

Outcomes in uncomplicated appendicitis are summarized in Table 5. Complication rates were low in both groups. SSI occurred in 6.7% of open and 2.0% of laparoscopic cases (p=0.330). Organ-space infection and ileus were not observed in the open group. Median hospital stay was shorter following laparoscopic appendectomy (three vs five days; p<0.001). Reoperation rates were rare and similar (1.3% vs 0; p=1.000).

**Table 5:** Postoperative outcomes in patients with uncomplicated appendicitis by surgical approach. N = total number of patients in each group; n = number of patients with the specified characteristic; SSI = surgical site infection; IAA = intra-abdominal abscess; IQR = interquartile range *Significant

Outcome	Laparoscopic (N=150)	Open (N=15)	p-value
Superficial SSI, n (%)	3 (2.0)	1 (6.7)	0.330
Deep SSI, n (%)	2 (1.3)	0 (0.0)	1.000
Organ-space SSI / IAA, n (%)	2 (1.3)	0 (0.0)	1.000
Postoperative ileus, n (%)	3 (2.0)	0 (0.0)	1.000
Postoperative bleeding, n (%)	2 (1.3)	0 (0.0)	1.000
Length of stay, days, median (IQR)	3 (2–4)	5 (4–6)	<0.001*
Reoperation ≤30 days, n (%)	2 (1.3)	0 (0.0)	1.000

**Figure 2:**
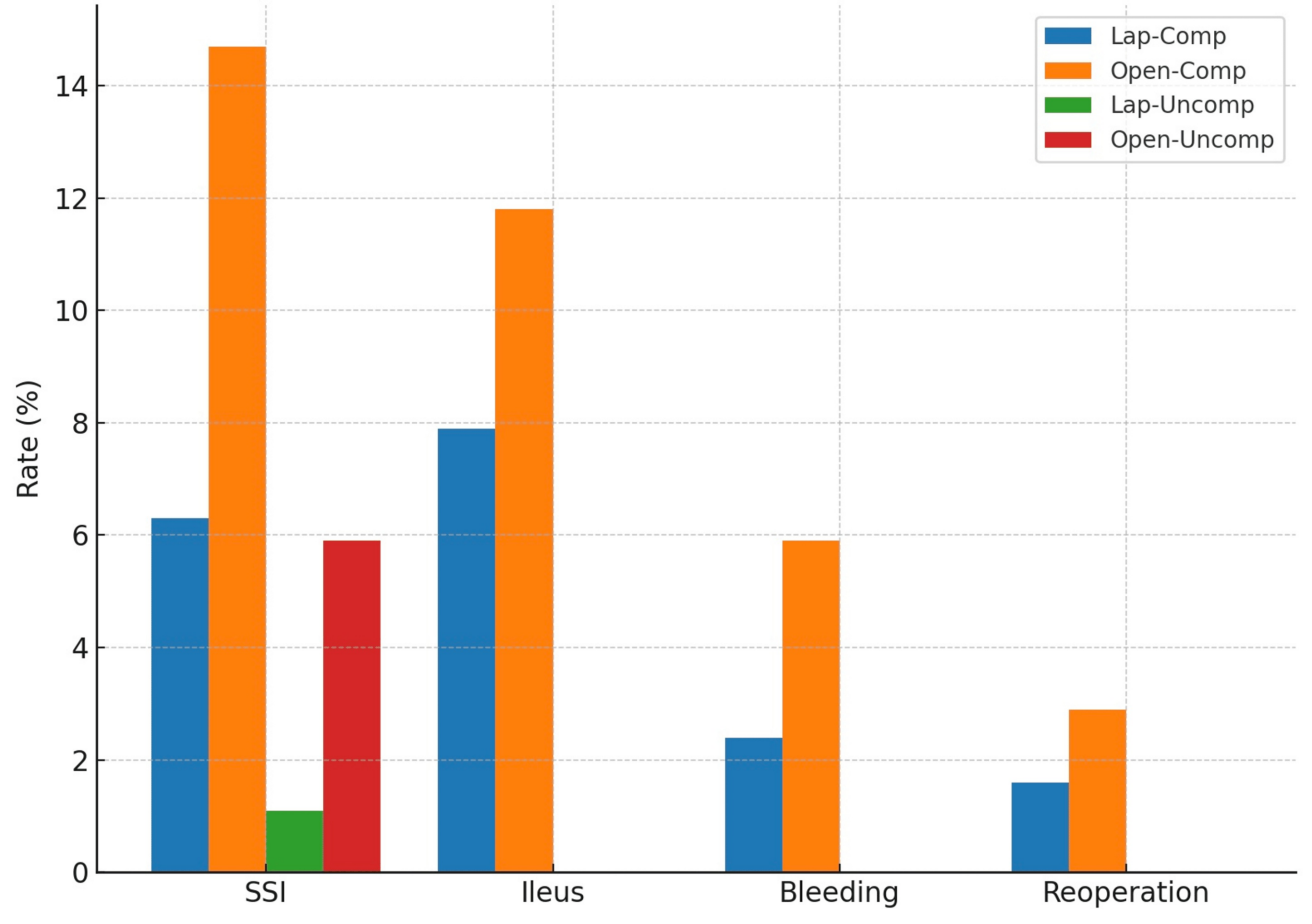
Postoperative complications after laparoscopic and open appendectomy in complicated and uncomplicated appendicitis. SSI = surgical site infection

Postoperative complications stratified by surgical approach and disease severity are shown in Figure 2. In complicated appendicitis, SSIs were more frequent in open cases (15.8% vs 8.2%), and ileus occurred in 26.3% of open versus 7.1% of laparoscopic cases. In uncomplicated appendicitis, SSIs occurred in 6.7% of open and 2.0% of laparoscopic cases, with no cases of ileus, bleeding, or reoperation observed in the open group.

**Figure 3:**
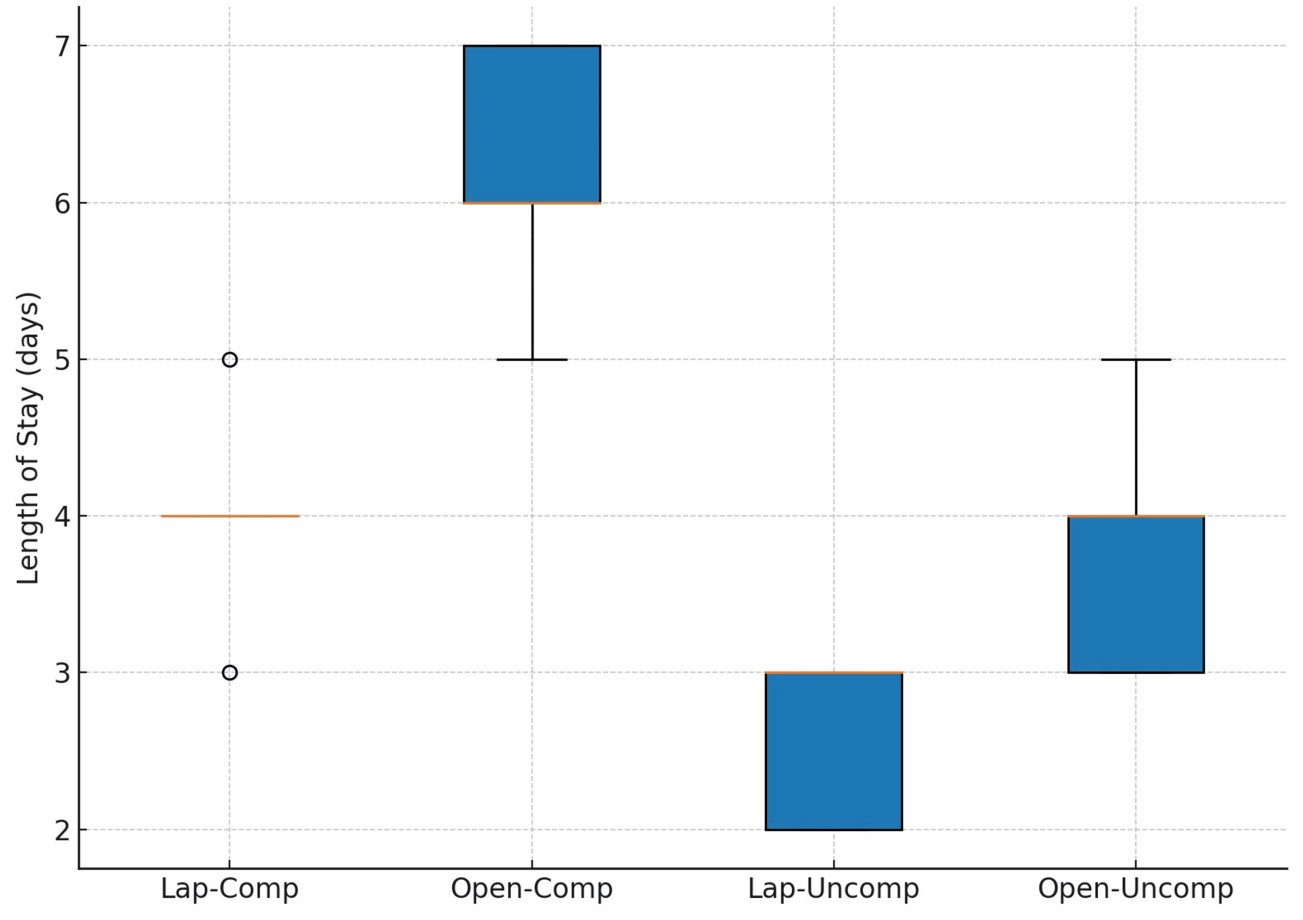
Length of hospital stay after laparoscopic and open appendectomy in complicated and uncomplicated appendicitis.

Length of postoperative hospital stay is illustrated in Figure 3. In complicated appendicitis, open cases had a median stay of seven days (IQR: 6-8) compared with five days (IQR: 4-6) for laparoscopic cases. In uncomplicated appendicitis, median stay was five days (IQR: 4-6) after open and three days (IQR: 2-4) after laparoscopic appendectomy.

## Discussion

This retrospective cohort study compared laparoscopic and open appendectomy in adults with both uncomplicated and complicated appendicitis over a two-year period. Our findings confirm that laparoscopic appendectomy is associated with favorable short-term outcomes compared with open surgery, particularly with respect to postoperative recovery and hospital stay. Patients undergoing laparoscopic appendectomy had significantly shorter hospitalization and lower rates of postoperative ileus, while wound-related complications trended lower, though differences did not always reach statistical significance. Importantly, these advantages persisted in complicated appendicitis, while outcomes in uncomplicated cases were uniformly excellent with either approach.

Several mechanisms can explain these findings. Laparoscopy limits bowel manipulation and reduces abdominal wall trauma compared with open incisions, thereby lowering the inflammatory stress response and promoting earlier return of gastrointestinal function. This likely underpins the reduced incidence of ileus observed in our study. Smaller incisions, improved visualization, and minimal tissue handling also reduce wound contamination and dead space, which may account for the trend toward fewer SSIs. These biologic advantages of laparoscopy are well described in recent studies [7-9] and help explain why minimally invasive approaches consistently result in shorter hospital stays across multiple settings.

Operative time was longer in the laparoscopic group, which is consistent with most series, particularly in centers where open appendectomy remains common. However, the trade-off is favorable: a modest increase in intraoperative duration offset by substantial downstream savings in recovery, complications, and length of stay. Similar observations have been made in Japanese and Egyptian studies, where mean operative times for laparoscopic appendectomy were approximately 58-65 minutes, compared with 45-50 minutes for open surgery. Despite the longer operative duration, laparoscopic patients recovered faster and returned to normal activity sooner [8,10]. From a systems perspective, the reduced length of stay after laparoscopy often offsets additional operating-room time and equipment costs [11].

When stratified by disease severity, the advantages of laparoscopy became more evident in complicated appendicitis. We found significantly lower rates of postoperative ileus and shorter hospital stay among patients treated laparoscopically, with no increase in reoperation or organ-space infection. The literature has reported concerns raised regarding a higher risk of intra-abdominal abscess following laparoscopic management of perforated appendicitis [5]. Indian studies provide local context: Shaikh et al. reported laparoscopic operative time of 47.2 ± 14.4 min vs open 36.9 ± 12.3 min but a significantly shorter hospital stay (3.7 vs 5.3 days; p<0.001) [12]. Similarly, Nath et al. found length of stay of 3.1 ± 0.5 days for laparoscopy versus 4.3 ± 0.7 days for open (p<0.0001), despite longer operative duration (55.1 vs 34.7 min) [13].

These findings are comparable to regional experiences from Egypt, where a prospective trial in complicated appendicitis showed similar length of stay between laparoscopic (5.1 days) and open (5.4 days) groups, but with fewer wound infections in the laparoscopic cohort [10]. In Japan, Shiihara et al. reported that more than 90% of complicated cases could be completed laparoscopically without increased morbidity [8].

However, accumulating evidence from recent meta-analyses and multicenter cohorts demonstrates that laparoscopy is safe and effective even in advanced disease. Zhang et al. found no significant difference in intra-abdominal abscess between laparoscopic and open approaches (3.8% vs 4.5%), while hospital stay was shorter in the laparoscopic group (4.5 vs 6.0 days) [9]. Gu et al.'s 2023 meta-analysis of perforated appendicitis similarly confirmed reduced wound infections and overall complications with laparoscopy, along with a pooled mean reduction in hospital stay of 1.3 days [14]. Rasuli et al. reported shorter hospitalization (4.2 vs 6.1 days) and fewer wound complications after laparoscopy, with comparable abscess rates [15].

In our study, the median length of stay (four to six days overall) was longer than the one to three days often reported in high-income settings. Our higher values likely reflect a greater proportion of complicated cases, as well as institutional practice patterns. Patients in our setting often receive prolonged intravenous antibiotics, remain admitted until bowel function is fully restored, and are discharged later due to conservative protocols and patient/family expectations. Similar extended stays have been described in Indian and regional cohorts, highlighting that hospital stay is influenced not only by surgical approach but also by institutional protocols and patient expectations [12,13].

Drain placement in our cohort was rare and limited to two complicated open cases. This selective use reflects evolving evidence and guidelines that discourage routine drainage after appendectomy for perforated or gangrenous appendicitis. Meta-analyses have shown that prophylactic drains do not reduce intra-abdominal abscess rates and may actually prolong hospital stay and increase overall morbidity [16]. A large retrospective cohort from 2022 similarly reported that, in complicated appendicitis managed laparoscopically, abdominal drains were associated with higher rates of complications and longer hospitalization without reducing abscess formation [17]. Our findings align with this evidence and support a restrictive approach to drains, reserving them for specific circumstances where source control is questionable.

From a systems perspective, these findings have broader implications. In many resource-limited settings, open appendectomy remains common due to constraints in laparoscopic capacity, cost, or expertise. However, multiple recent studies, including those from Egypt and Asia [8,10], have demonstrated that the benefits of laparoscopy persist even in such environments. Shorter hospital stays reduce bed occupancy, lower indirect costs, and improve patient turnover, making laparoscopy a cost-effective option when considered across the continuum of care. As laparoscopic skills and infrastructure expand globally, the case for laparoscopy as the preferred standard strengthens further.

Our study has several strengths. It includes a consecutive adult cohort over a clearly defined two-year period, with subgroup analysis by disease severity. Standardized definitions were applied for complicated appendicitis and surgical site infections, ensuring consistent outcome classification. Relevant endpoints, including operative details, complications, and length of stay, were comprehensively captured. Moreover, the findings are directly applicable to real-world surgical practice, reflecting outcomes from a typical general surgical unit.

Limitations must also be acknowledged. The retrospective design introduces risks of bias, and causal relationships cannot be established. Selection bias is possible, as surgeons may preferentially choose open appendectomy in more advanced cases, although stratified analyses were performed to address this. The relatively small number of open cases limited statistical power for uncommon outcomes such as organ-space infection and reoperation, raising the risk of type II error. Comorbidities and ASA classification were not analyzed, which could influence both operative choice and outcomes. Long-term complications such as adhesive obstruction, incisional hernia, and chronic pain were not assessed. Finally, this was a single-center experience, and results may not generalize to institutions with different patient populations or perioperative protocols.

Despite these limitations, the implications are important. Laparoscopic appendectomy was consistently associated with faster recovery and lower morbidity, even in complicated appendicitis. These results support current international guidelines, which recommend laparoscopy as the standard of care for both uncomplicated and complicated disease [11]. Open appendectomy remains necessary in selected circumstances - for example, when laparoscopic visualization is inadequate or equipment is unavailable - but should no longer be considered the default approach.

Future studies should build on this work. Large multicenter prospective cohorts or registry-based analyses with standardized data collection are needed to validate these findings, particularly in complicated appendicitis. Economic evaluations comparing not just operative costs but total episode-of-care expenses (including readmissions, complications, and patient-reported outcomes) will provide valuable insight for policymakers in resource-constrained settings. Research into optimal perioperative strategies - including antibiotic duration and the role of selective drainage - may refine management further. Finally, incorporating patient-centered outcomes such as time to return to work, long-term quality of life, and pain will ensure that evidence translates into meaningful improvements for patients.

## Conclusions

This two-year retrospective cohort study supports the safety and efficacy of laparoscopic appendectomy in both uncomplicated and complicated appendicitis. Compared with open surgery, laparoscopy was associated with shorter hospital stay and lower rates of postoperative ileus, with comparable rates of infection and reoperation. Open appendectomy remains necessary in selected cases, but the laparoscopic approach should be considered the preferred standard where expertise and resources allow. Future prospective multicenter studies with larger sample sizes are warranted to further clarify outcomes, particularly in complicated appendicitis.
